# Circadian regulation of chemotherapy-induced peripheral neuropathic pain and the underlying transcriptomic landscape

**DOI:** 10.1038/s41598-020-70757-w

**Published:** 2020-08-14

**Authors:** Hee Kee Kim, Sun-Yeul Lee, Nobuya Koike, Eunju Kim, Marvin Wirianto, Mark J. Burish, Kazuhiro Yagita, Hyun Kyoung Lee, Zheng Chen, Jin Mo Chung, Salahadin Abdi, Seung-Hee Yoo

**Affiliations:** 1 Division of Anesthesiology, Critical Care and Pain Medicine, Department of Pain Medicine, The University of Texas MD Anderson Cancer Center, Houston, TX 77030 USA; 2 Department of Biochemistry and Molecular Biology, The University of Texas Health Science Center At Houston, 6431 Fannin St., Houston, TX 77030 USA; 3 Department of Anesthesiology and Pain Medicine, Chungnam National University Hospital, Daejeon, South Korea; 4 Department of Physiology and Systems Bioscience, Graduate School of Medical Science, Kyoto Prefectural University of Medicine, Kyoto, Japan; 5 Department of Neurosurgery, The University of Texas Health Science Center at Houston, 6400 Fannin St., Houston, TX 77030 USA; 6 Department of Pediatrics, Baylor College of Medicine, Neurological Research Institute, Texas Children’s Hospital, Houston, TX 77030 USA; 7 Department of Neuroscience, Cell Biology and Anatomy, University of Texas Medical Branch, Galveston, TX USA

**Keywords:** Circadian rhythms and sleep, Neurophysiology

## Abstract

Growing evidence demonstrates circadian rhythms of pain hypersensitivity in various chronic disorders. In chemotherapy-induced peripheral neuropathy (CIPN), agents such as paclitaxel are known to elicit chronic neuropathic pain in cancer patients and seriously compromise their quality of life. Here, we report that the mechanical threshold for allodynia in paclitaxel-treated rats exhibited a robust circadian oscillation, reaching the nadir during the daytime (inactive phase). Using Per2::LucSV circadian reporter mice expressing a PER2::LUC fusion protein, we isolated dorsal root ganglia (DRG), the primary sensory cell body for peripheral nerve injury generated hypersensitivity, and monitored ex vivo reporter bioluminescence. We observed strong circadian reporter rhythms in DRG neurons which are highly entrainable by external cues. Paclitaxel treatment significantly lengthened DRG circadian periods, with little effects on the amplitude of oscillation. We further observed the core protein BMAL1 and PER2 in DRG neurons and satellite cells. Using DRG and dorsal horn (DH; another key structure for CIPN pain response) tissues from vehicle and paclitaxel treated rats, we performed RNA-sequencing and identified diurnal expression of core clock genes as well as clock-controlled genes in both sites. Interestingly, 20.1% and 30.4% of diurnal differentially expressed genes (DEGs) overlapped with paclitaxel-induced DEGs in the DRG and the DH respectively. In contrast, paclitaxel-induced DEGs displayed only a modest overlap between daytime and nighttime (*Zeitgeber* Time 8 and 20). Furthermore, paclitaxel treatment induced de novo diurnal DEGs, suggesting reciprocal interaction of circadian rhythms and chemotherapy. Our study therefore demonstrates a circadian oscillation of CIPN and its underlying transcriptomic landscape.

## Introduction

Numerous vital processes in our body display time-of-day dependent oscillation. Such daily rhythms of tissue-specific and systemic functions are driven by the endogenous circadian clock where cellular oscillators, containing interlocked transcriptional-translational loops composed of positive (CLOCK/NPAS2, BMAL1, RORs) and negative (PERIOD1/2 (PER1/2) and CRYPTOCHROME1/2 (CRY1/2)) factors^1^, are orchestrated by the central pacemaker in the suprachiasmatic nuclei (SCN) in the hypothalamus. Pain is a fundamental challenge in disease and intervention, and increasing evidence has illustrated the prevalence of circadian oscillation in pain response. For example, episodic pain attacks in cluster headache display pronounced circadian patterns, where 82% of patients have a 15–180 min headache at the same time each day^2,3^. Diseases with continuous pain also exhibit circadian patterns. Neuropathic pain can be triggered by various insults to both the central and peripheral nervous systems, and recent studies have shown circadian rhythms of neuropathic pain intensity under several conditions^4–6^. In a pioneering study using mice with sciatic nerve injury, diurnal rhythms of mechanical allodynia were found to be mediated by induction of extracellular ATP release from spinal astrocytes by the circadian hormone glucocorticoid^5^.

Chemotherapy-induced peripheral neuropathy (CIPN) is a dose-limiting adverse effect that can take place at any time during the course of treatment or even after its termination^7^. CIPN affects up to 80% of cancer patients treated with chemotherapy agents^8–10^ leading to significant decline in the overall quality of life in cancer patients^11,12^. As a severe adverse effect of their cytostatic pharmacotherapy, the dorsal root ganglia (DRG) of sensory neurons and the dorsal horn (DH) of spinal cords in the pain processing circuitry suffer neurotoxic damage, which can lead to sensory ataxia, paresthesia, dysesthesia, mechanical allodynia, cold allodynia^9^, and ultimately peripheral pain in the distal extremities in a symmetrical glove and stocking distribution^13^.

Despite severe adverse effects of CIPN on the quality of life, the underlying pathophysiology remains elusive^14,15^. The taxane class of antineoplastic drugs, including paclitaxel, docetaxel and cabazitaxel, inhibit normal cycles of microtubule depolymerization and repolymerization, and are known to cause high incidence rates of CIPN, especially paclitaxel^16^. As a result, paclitaxel-induced CIPN is a widely used laboratory model of neuropathic pain observed in CIPN patients. Recent studies have shown a molecular link of paclitaxel-induced CIPN with the circadian clock. For example, the core clock gene *Bmal1* was found to serve as a tumor suppressor for tongue squamous cell carcinoma, and tumor cells with increased *Bmal1* expression showed increased sensitivity to paclitaxel^17^. Additionally, paclitaxel altered mRNA expression of several circadian genes (*Period 3*, *Dec1*, and *Dec2*) in several cancer cell lines^18,19^. However, circadian regulation of paclitaxel-induced CIPN has not been well studied, and the underlying molecular pathways are poorly understood.

In the current study, a rat model of CIPN was found to demonstrate circadian oscillations of mechanical pain hypersensitivity, and RNA-sequencing discovered a large overlap of circadian and pain response genes in both the DRG and the DH.

## Results

### Paclitaxel-induced neuropathic pain behavior in rats

**Figure 1:**
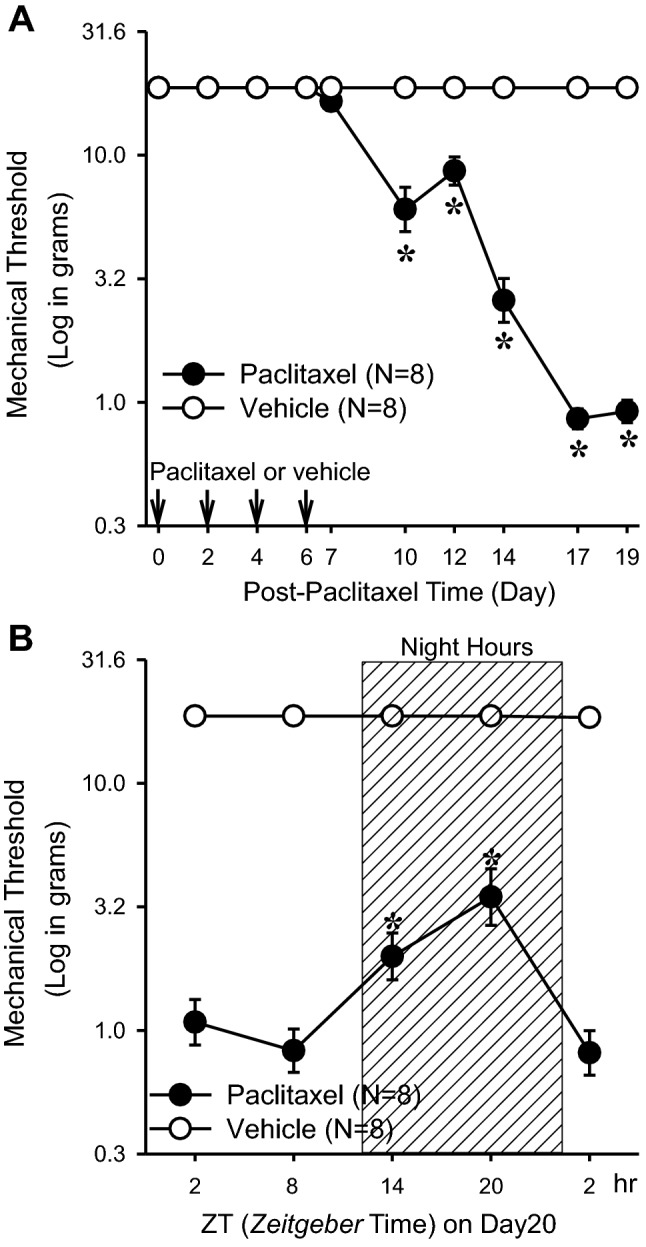
Effects of circadian rhythms in paclitaxel-induced neuropathic pain in rats. (**A**) Paclitaxel (PAC, 2 mg/kg, n = 8) or vehicle (4% dimethyl sulfoxide and 4% Tween 80 in saline, 1 ml/kg, n = 8) was injected intraperitoneally on four alternate days (days 0, 2, 4, and 6), and the mechanical threshold was measured. The asterisks indicate values that are significantly different (P < 0.05) from the corresponding values for the saline group as determined by a two-way repeated-measures analysis of variance with one repeated factor (time) followed by the Tukey post hoc test. (**B**) On day 20 after the first paclitaxel (n = 8) or vehicle (n = 8) injection, the mechanical threshold was measured at 2, 8, 14, 20, and 2 h after light-on. The asterisks indicate values that are significantly different (*p* < 0.05) from the 8 h after light-on paclitaxel group as determined by a one-way repeated-measures analysis of variance with one repeated factor (time) followed by the Tukey post hoc test. The data are presented as means with error bars represent SEM (n = 8).

To investigate a functional link between circadian rhythms and CIPN, we employed a well-established peripheral nerve injury model, namely paclitaxel-treated rats, as previously described^20^. Rats were housed in the normal 12:12 h light:dark condition and injected with paclitaxel (2 mg/kg on days 0, 2, 4, and 6, total 8 mg/kg) or vehicle (dimethyl sulfoxide and Tween 80 in saline, same volumes and intervals as in paclitaxel-injected rats). By von Frey assays performed in the morning, we found that paclitaxel significantly decreased mechanical thresholds from 18 g on day 0 to 1.1 g on day 20, whereas the vehicle did not change the mechanical threshold (18 g) during the experimental duration (Fig. 1A). Next we examined the circadian pattern of CIPN on day 20. Specifically, we performed von Frey behavioral testing at *Zeitgeber* time (ZT) 2, 8 and 26 under normal light and ZT14 and ZT20 under red light (ZT 0 corresponds to light-on during the 12:12 light:dark cycle). In rats treated with paclitaxel, pain response showed a significant circadian oscillation. The mechanical threshold was increased from 0.8 g at ZT8 to 3.5 g at ZT20, suggesting lowest (trough) and highest (peak) pain tolerance at ZT8 (daytime) and ZT20 (nighttime) respectively (Fig. 1B). In contrast, vehicle treated rats did not show significant mechanical threshold changes. These results indicate a circadian rhythm of neuropathic pain hypersensitivity in a chemotherapy-induced rat model.

### Real-time circadian bioluminescence rhythms in ex vivo DRG cultures from Per2::LucSV circadian reporter mice

**Figure 2:**
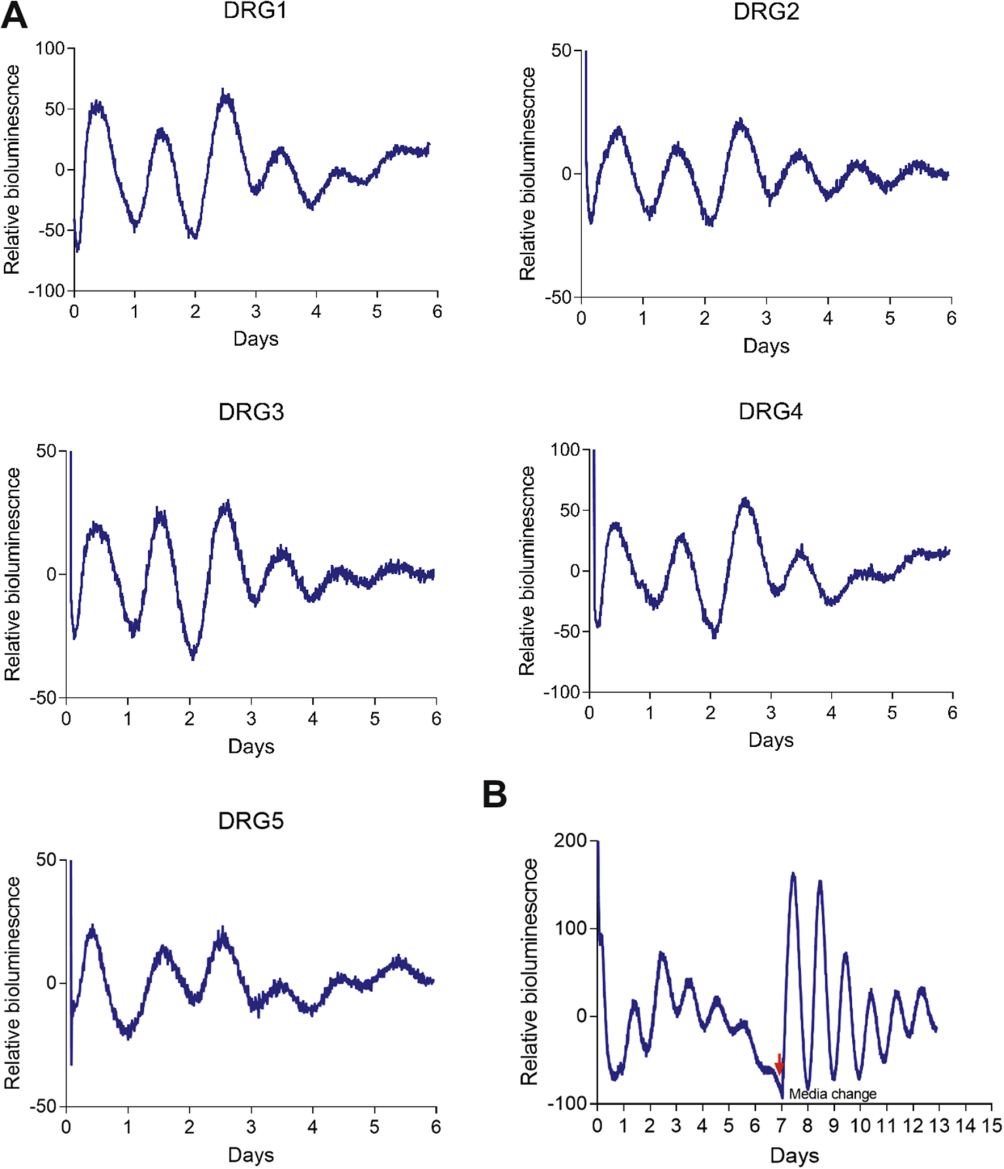
Real-time analysis of circadian expression of PER2::LUC in ex vivo DRG cultures. (**A**) Representative records of bioluminescence (LumiCycle Analysis, v. 3.0002, Actimetrics) showing circadian profiles of PER2::LUC expression from DRG1 to DRG5 isolated from Per2::LucSV mice (n = 48, 8–9 each for DRG L1–L5). DRGs were explanted before light off. (**B**) Circadian oscillation of DRG explants persist rhythmicity after media change. After day 7 of culture, media were replaced and circadian oscillation was reinitiated in L4 DRG explants (n = 3).

We previously produced Per2::Luc and Per2::LucSV reporter mice that express PER2::LUC fusion proteins under the endogenous mouse *Per2* promoter^21,22^, and these mice have been widely used to monitor circadian dynamics with high temporal resolution in different tissues and revealed tissue-specific clocks with distinct phases and periods^21,22^. Compared with Per2::Luc, Per2::LucSV mice demonstrate enhanced bioluminescence oscillation and are thus ideally suited for detecting oscillations in small tissues or cell populations^22–24^. Here, we used Per2::LucSV mice to derive ex vivo DRG cultures for circadian bioluminescence monitoring. Lumbar DRG cultures from L1 to L5 showed robust circadian oscillations of PER2::LUC bioluminescence with an average period of 24.2 h (Fig. 2A). Previously we observed dampening of PER2::LUC bioluminescence oscillation as a result of desynchronization of individual oscillators especially in peripheral tissues, and media change was able to re-synchronize oscillators and recover robust circadian oscillation^21^. Interestingly, after the initial 6-day circadian bioluminescence oscillation, media change on day 7 led to a persistent, self-sustained PER2::LUC rhythm with a circadian amplitude (the difference between peak and trough values) significantly greater than that before the synchronization (Fig. 2B). These results indicate robust circadian oscillations in DRG neurons which are sensitive to entraining signals.

### Paclitaxel effects on ex vivo DRG culture circadian rhythms

**Figure 3:**
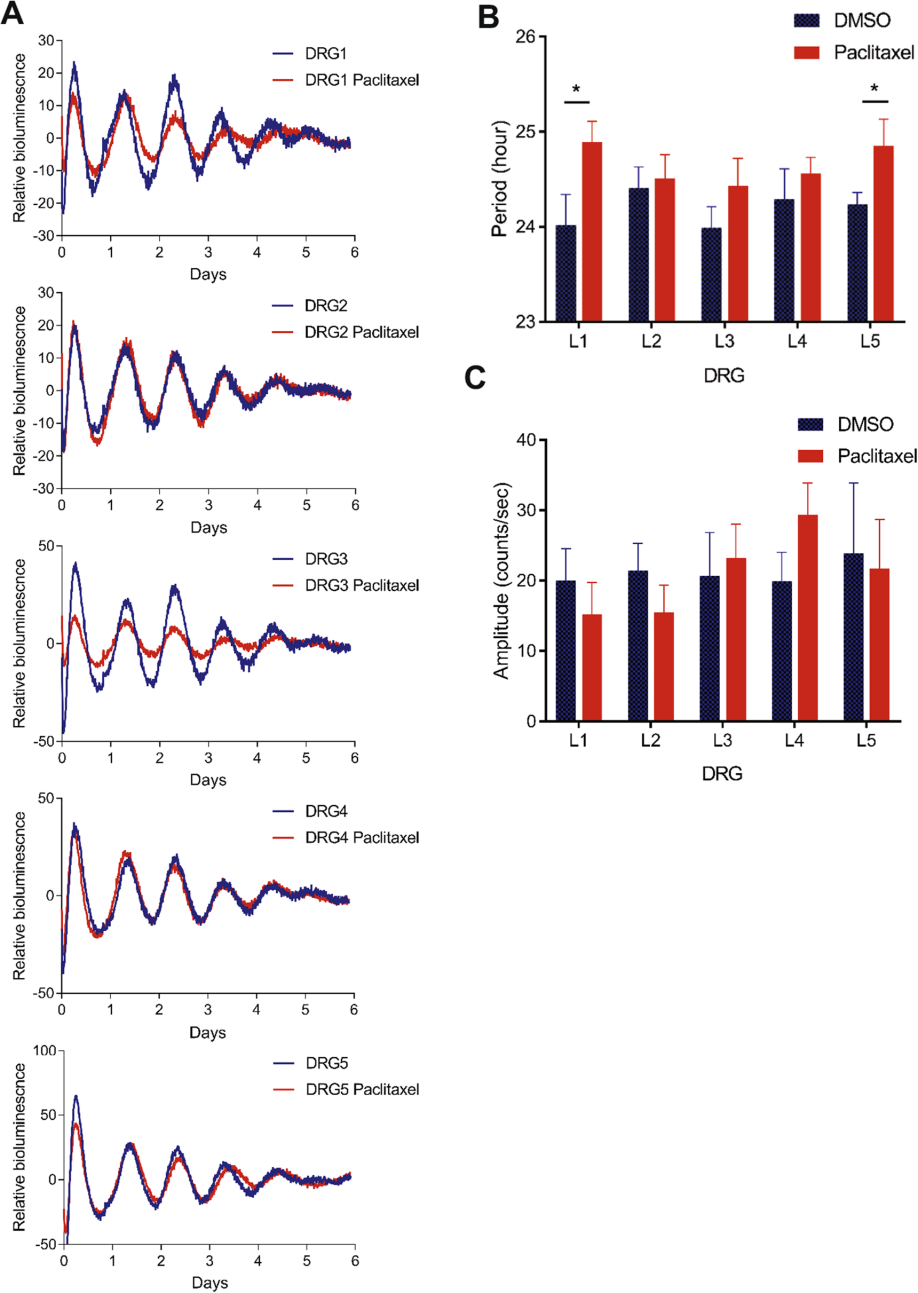
Effect of paclitaxel on circadian rhythms of DRG ex vivo cultures. Real-time circadian bioluminescence was measured from paclitaxel and vehicle treated DRG1–DRG5 ex vivo cultures. (**A**) Representative records of bioluminescence showing circadian rhythm of PER2::LUC expression from vehicle and paclitaxel treated DRG (n = 7–8). Blue and red traces represent PER2::LUC reporter rhythms from vehicle and paclitaxel (20 μM) groups respectively. (**B**) DRG period comparisons are shown. Period was calculated by the LM fit (damped sin) method (LumiCycle analysis v.3.0002, Actimetrics). L1–L5 DMSO group vs. Paclitaxel group *t* test: L1 and L5 DMSO vs. paclitaxel t-test: *p < 0.05. (**C**) Relative fold amplitudes of vehicle and paclitaxel treated tissues were calculated by the LM fit (damped sin) method. Error bars represent SEM (n = 7–8).

Next we examined effects of paclitaxel on circadian reporter rhythms from ex vivo DRG cultures. DRGs were isolated from Per2::LucSV mice and cultured with DMSO- or paclitaxel-containing recording media. Individual DRG cultures (L1–L5) showed periods in the circadian range (~ 24 h) (Fig. 3A,B), and paclitaxel treatment led to varying degrees of period lengthening compared to DMSO controls, with statistically significant effects observed for L1–L5 (p < 0.05) (Fig. 3B). The neurotoxic effect of paclitaxel did not appear to adversely impact circadian oscillatory amplitude, namely the difference between peak and trough (Fig. 3C). These results suggest that acute paclitaxel treatment affects the period, but not the amplitude, of the molecular oscillator.

### Expression of core clock components BMAL1 and PER2 in the DRG

**Figure 4:**
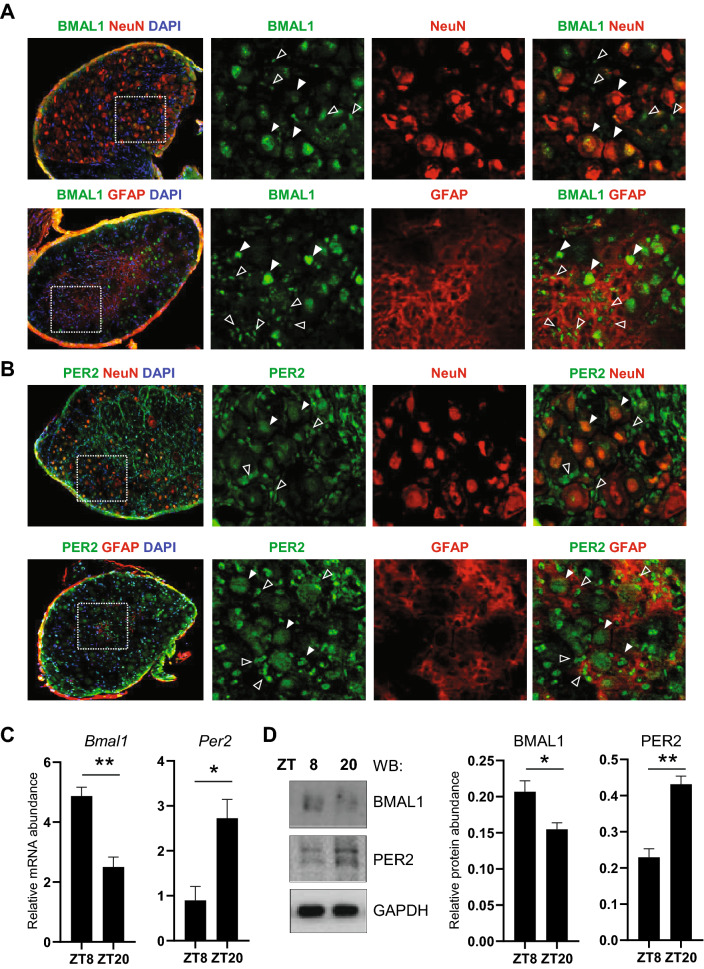
Expression of core clock proteins PERIOD2 and BMAL1 in DRG neurons and satellite cells. (**A**) Representative images of BMAL1 expression in the DRG of WT mice at ZT20. BMAL1 (green), GFAP (red), NeuN (red) and DAPI (blue). (**B**) Representative images of PER2 expression in DRG of WT mice at ZT20. PER2 (green), GFAP (red) and NeuN (red) and DAPI (blue) (N = 3). Closed triangle: neuron, open triangle: satellite cell. (**C**) Real-time qPCR analysis of *Bmal1* and *Per2* expression in the DRG. Error bars represent SEM (n = 3) *p < 0.05, **p < 0.01, unpaired *t*-test. (D) Western blotting was performed using total DRG protein extracts with indicated antibodies. Error bars represent SEM (n = 3), *p < 0.05, **p < 0.01, unpaired *t*-test.

Next, we examined the expression of the core clock proteins BMAL1 and PER2 in the mouse DRG by immunostaining. First, we double-stained DRG tissue sections collected at ZT20 with antibodies against BMAL1, the neuronal marker NeuN and the glial marker GFAP. The results revealed BMAL1 expression in the nuclei of neurons with large or small diameters and satellite glial cells where it co-localized with NeuN and GFAP staining signals respectively, suggesting that BMAL1 is expressed in both neurons and satellite cells (Fig. 4A). Next, we used PER2 antibody to detect PER2 expression in the DRG. Consistent with BMAL1 staining results, PER2 expression was also detected in both neurons and satellite glial cells, with stronger expression found in the latter (Fig. 4B). We next examined mRNA and protein expression of *Bmal1* and *Per2* using DRG tissues isolated from Per2::LucSV mice at ZT8 and ZT20. Real-time qPCR and immunoblotting analyses revealed diurnal expression of *Bmal1* and *Per2* at the transcript (Fig. 4C) and protein (Fig. 4D) levels in mouse DRG. Taken together, these results reveal oscillatory clock protein expressions in the DRG.

### Diurnal transcriptome profiling of CIPN

**Figure 5:**
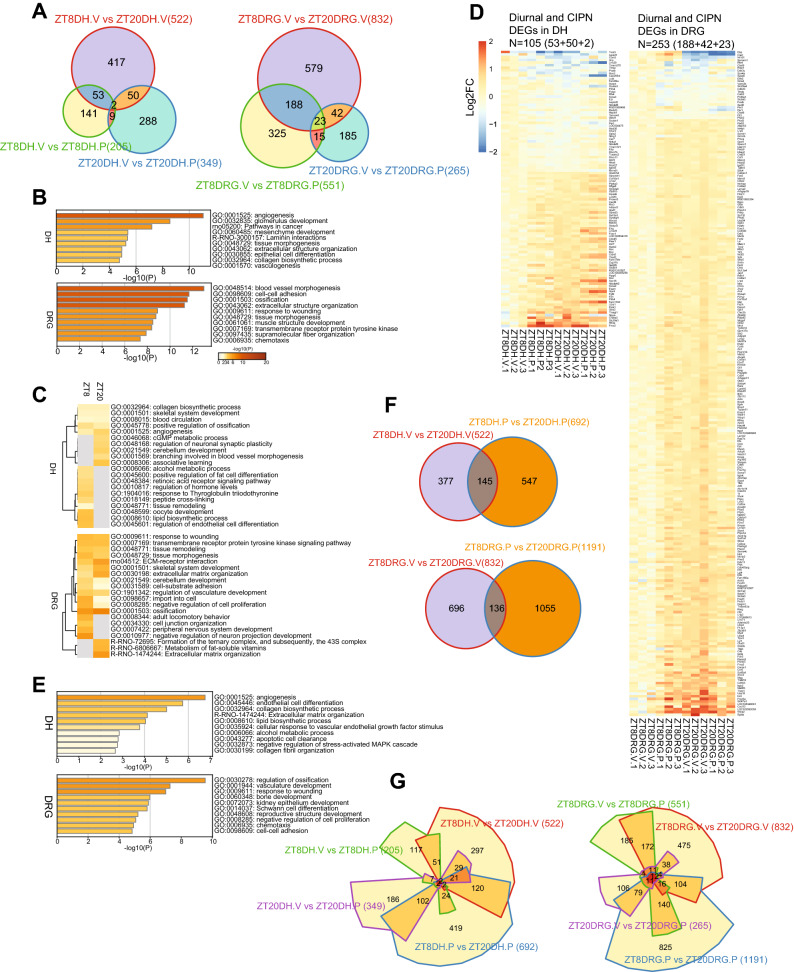
Circadian transcriptomic landscape in the DRG and the DH. The differentially expressed genes were determined by using DESeq2 with thresholds of *p* < 0.05 and fold change > 1.2. (**A**) Venn diagrams of differentially expressed genes in vehicle and paclitaxel treated DH (left panel) and DRG (right panel). “ZT8DH.V vs. ZT20DH.V” (522) and “ZT8DRG.V vs. ZT20DRG.V” (832): Red circles indicate differentially expressed genes (DEGs) at two circadian time points (ZT8 and ZT20) in DH and DRG respectively. “ZT8DH.V vs. ZT8DH.P” (205) indicates paclitaxel-induced DEGs at ZT8 (green circle) and “ZT20DH.V vs. ZT20DH.P” (349) indicates paclitaxel-induced DEGs at ZT20 (blue circle) in the DH (left panel). “ZT8DRG.V vs. ZT8DRG.P” (551) indicates paclitaxel induced DEGs at ZT8 (green circle) and “ZT20DRG.V vs. ZT20DRG.P” (265) indicates paclitaxel induced DEGs at ZT20 in DRG (blue circle). (**B**) Heat map showing the top enrichment clusters by Metascape analysis (https://metascape.org/) using diurnal DEGs in the DH and the DRG. (**C**) Heat map showing the top enrichment clusters by Metascape analysis using paclitaxel-induced CIPN DEGs in the DH and the DRG at two circadian time points (ZT8 and ZT20). (**D**) Heat map view of DEGs at the intersection of diurnal genes and CIPN DEGs in the DH and the DRG. Each gene is represented as a horizontal line ordered vertically by log2 fold change in expression level at ZT20 with vehicle treatment relative to ZT8. (**E**) Heat map showing the top enrichment clusters by Metascape analysis using the overlapped genes between diurnal DEGs and paclitaxel-induced CIPN DEGs. Discrete color scale represents statistical significance, while gray color indicates a lack of significance. (**F**) Venn diagrams of diurnal DEGs in vehicle and paclitaxel-induced DEGs in DH (upper panel) and DRG (lower panel). Red circle: diurnal DEGs, blue circle: paclitaxel induced diurnal DEGs. (**G**) Chow-Ruskey diagrams showing the four-way overlap of diurnal DEGs, CIPN DEGs (ZT8 and ZT20) and paclitaxel-induced DEGs in both tissues. The boundaries for each DEGs are color-coded: diurnal DEGs (red), CIPN DEGs at ZT8 (green) and ZT20 (purple) and paclitaxel-induced DEGs (blue). The domain areas are proportional to the number of DEGs. The Venn diagrams, heat maps for gene expression and Chow-Ruskey diagrams were prepared with R version 3.6.3 (https://github.com/js229/Vennerable, https://CRAN.R-project.org/package=pheatmap).

To survey the global diurnal transcriptome landscape in both the DH and the DRG in response to CIPN, we performed RNA-seq using RNA samples from tissues isolated from vehicle and paclitaxel treated rats at ZT8 and ZT20 (daytime and nighttime, respectively). We found diurnal expression of a number of core clock genes in both tissues (Fig. S1, Tables S1 and S2), including *Per1, Cry1*, *Nr1d1*, *Nr1d2* and *Dbp*. Note that clock genes are expressed with different phases and examination of additional time points is likely required to identify additional clock genes with differential expression. Gene expression comparison between the two time points from vehicle treated animals revealed 522 and 832 diurnal DEGs (differential expressed genes) in the DH and the DRG respectively (Fig. 5A). Functional enrichment analysis of gene clusters by Metascape^25^ showed strong enrichment of angiogenesis and glomerulus development pathways in the DH and muscle contraction, blood vessel morphogenesis and cell–cell adhesion pathways in the DRG (Fig. 5B). Interestingly, we found diurnal DEGs shared between the DRG and the DH, including the membrane transporter solute carrier (Slc) superfamily (primarily involved small molecules uptake into cells) and the ATP-dependent efflux ABC transporters^26^ (Tables S1 and S2). Ion channels, including voltage-gated sodium channels (Nav), voltage-gated potassium channels (Kv), and transient receptor potential channels (TRP), are well-known contributors to the development of CIPN^27–29^. Genes encoding Nav, Kv, and TRP were found to display diurnal expression pattern in both tissues (Tables S1 and S2).

Next, to identify CIPN-related gene expression changes as a function of circadian time, we compared DEGs from vehicle- and paclitaxel-treated tissues at ZT8 and ZT20. DH and DRG showed distinct sets of DEGs at ZT8 and ZT20 with very modest numbers of shared genes between the two time points, indicating strong time-of-day effects on CIPN transcriptomes (Fig. 5A, Tables S3 and S4). Functional enrichment analysis revealed enrichment of distinct pathways at ZT8 and ZT20. In the DH, genes involved in lipid biosynthesis and endothelial cell differentiation were enriched at ZT8, and those related to associative learning and neuronal synaptic plasticity were enriched at ZT20. In the DRG, genes for peripheral nervous system development and inhibition of neuronal projection development were enriched at ZT8, and those for extracellular matrix organization and ECM-receptor interaction were enriched at ZT20 (Fig. 5C). Among the diurnal DEGs, approximately 20% (53 + 2 + 50) and 30% (188 + 23 + 42) genes are shared with CIPN DEGs in the DH and the DRG respectively (Fig. 5D, Tables S5 and S6). This result indicates broad circadian regulation of CIPN DEGs, suggesting a cellular basis for the observed circadian pain behavior. Functional enrichment analysis showed strong enrichment of angiogenesis and lipid biosynthesis genes in the DH, and genes for ossification regulation and Schwann cell differentiation in the DRG (Fig. 5E).

To further evaluate the effect of paclitaxel on the circadian transcriptome, we identified paclitaxel-induced diurnal DEGs (Fig. 5F, blue circles) similarly as for vehicle-dependent diurnal DEGs in Fig. 5A (red circles), and compared these two sets of diurnal DEGs (vehicle vs. paclitaxel). We identified sizable de novo diurnal DEGs generated by paclitaxel treatment in both the DH (547) and the DRG (1,055). The overlaps of diurnal DEGs between vehicle and paclitaxel treatments are small (145 and 136 for DH and DRG respectively; Fig. 5F) and core clock genes including *Bmal1*, *Cry1*, *Nr1d1*, *Nr1d2*, and *Dbp* retained diurnal oscillation following paclitaxel treatment as well as genes encoding Kv and Nav channels (Fig. 5F). Furthermore, *Oprm1* (mu-opioid-receptor) and *Oprd1* (opioid receptor delta 1) in the DRG, and *Adora2a* and *IGF1* in both the DH and the DRG showed diurnal expression following paclitaxel treatment, suggesting that paclitaxel treatment can reciprocally reprogram the circadian transcriptome (Tables S7 and S8). Functional analysis showed enrichment of heart and endoderm development genes in the DH and eukaryotic translation initiation and synapse organization in the DRG (Fig. S2). Next we compared DEGs of all four groups, namely diurnal, CIPN (ZT8 and ZT20), and paclitaxel induced diurnal DEGs in both tissues (Fig. 5G, Fig. S3). CIPN and paclitaxel-induced de novo diurnal DEGs showed modest overlap (Fig. 5G), suggesting paclitaxel treatment generates distinct transcriptome signatures independent from diurnal and CIPN transcriptomes.

DRG gene regulation plays pivotal roles in neuropathic pain genesis^30–32^, and RNA sequencing studies have elucidated the molecular signature underlying neuropathic pain induced by nerve injury^33–40^. We compared our CIPN induced DRG DEGs with results from nerve injury induced neuropathic pain^38,41^. To our surprise, the two different pain models showed a small number of shared DEGs (Fig. S4, Table S9), suggesting pain-specific transcriptomic remodeling.

We further compared our data to pain-related gene sets based on previous studies^42–44^ and databases (https://painresearchforum.org/resources/pain-gene-resource, humanpaingenetics.org/hpgdb/). Depending on circadian time and treatment, this analysis identified a group of overlapping genes functioning in pain and circadian rhythms (Table S10). Many core clock genes were found, including *Cry1*, *Dbp*, *Nr1d1*, *Nr1d2*, *Per1*, *Per2*, *Per3,* and *Rora*. Several well-known pain genes were also identified, including *Oprm1* (encoding the mu 1 opioid receptor) and *P2rx7* (encoding the purinergic receptor P2X 7). Other opioid receptor genes were observed, namely *Oprd1* and *Oprk1* (encoding the delta 1 and kappa 1 opioid receptors respectively). Finally our analysis also revealed *Trpv3,* but not the *Trpv1, Trpv2*, or *Trpv4* genes, and two genes involved in migraine, namely *Atp1a2* and *Scn1a* (mutations of which are responsible for familial hemiplegic migraine types 2 and 3 respectively)^45^. These results are consistent with an extensive crosstalk between circadian and pain pathways.

## Discussion

In the current study, we demonstrated a robust circadian rhythm of CIPN using paclitaxel-treated rats. Of note, we observed a low pain tolerance in rats during the day (inactive phase), which correlates with the clinical observation of low mechanical thresholds at night in humans with neuropathic pain^46,47^. We then performed ex vivo cultures of the peripheral ganglion DRG from Per2::LucSV reporter mice and showed cell-autonomous circadian oscillation. Paclitaxel treatment of ex vivo DRG cultures showed period lengthening effects without altering amplitude, indicating a surprisingly resilient circadian oscillation against the cytotoxicity of paclitaxel. DRG neurons and satellite cells both expressed the core clock proteins BMAL1 and PER2. From DH and DRG RNA-sequencing, we showed that both tissues have robust diurnal oscillations in key gene clusters including the membrane transporter solute carrier (Slc) superfamily, ATP-dependent efflux ABC transporters, and ion channels (Nav, Kv, TRP). Analysis of CIPN DEGs showed distinct gene sets at two circadian time points which strongly overlap with clock-controlled genes. Together, our study demonstrates robust circadian regulation of paclitaxel CIPN and the underlying gene expression network.

CIPN is a common side effect in both older and newer chemotherapeutic agents, and there is an urgent need to improve both prevention and treatment of CIPN^48^. Paclitaxel is a widely used first-line drug for breast cancer. While it cannot penetrate the blood–brain barrier and thus does not accumulate in the central nervous system, unfortunately it can damage the peripheral nervous system including the DRG, leading to pain development^49–51^. In the current study, we show a clear circadian pattern of paclitaxel CIPN, suggesting the clock as a neurophysiological mechanism regulating this pain response. This result is consistent with previous findings of circadian pain responses in various other peripheral neuropathies^4^.

Compared with qPCR analysis, circadian reporter bioluminescence monitoring in ex vivo cultures has superior temporal resolution and is also well suited for studies of drug effects on cellular oscillators. Extending previous studies of clock gene expression in the DRG^52,53^, we performed DRG ex vivo cultures and revealed that L1–L5 DRG populations showed robust circadian reporter rhythms where rhythmic amplitude can be strongly induced by media change, a simple synchronizing cue. Extending the circadian reporter results, we further provide evidence for diurnal expression of the endogenous clock proteins BMAL1 and PER2 in both neuron and satellite cells in the DRG. Furthermore, paclitaxel was found to lengthen the circadian period to varying degrees, with the most significant effects in L1 and L5. These observations demonstrate that paclitaxel directly modulates cellular oscillators at the DRG, and that the DRG clocks are both amenable to entrainment and their robustness is resilient to the cytotoxic effects of paclitaxel.

Circadian oscillators drive tissue-specific transcriptomic expression^54–57^. The observation that paclitaxel can modulate circadian oscillators in the DRG prompted us to investigate its transcriptomic landscape. Paclitaxel induces several events in the DRG including: 1. Accumulation of immune cells such as macrophage and polymorphonuclear cells; 2. An increase in calcium channel subunits in the DRG^58–60^; 3. An increase in the expression of phospholipase A2, chemokine ligand 21b, complement components 1 and 3, and matrix metalloproteinase 3^61^; and 4. An increase of inflammatory cytokines such as TNF-α, IL-1, and IL-6^62^. These events may be responsible for changes in pain behavior. From our RNA-seq analysis, CIPN DEGs in the DH and the DRG largely overlap with these pathways as revealed by Metascape pathway analysis (Figs. S5 and S6). We also included DH tissues for transcriptomic profiling due to its pivotal role, in conjunction with the DRG, in the pain response. The results indicate a broad diurnal transcriptome in the DH, consistent with a fundamental role of circadian rhythms in CIPN.

A close examination of the transcriptomic landscape revealed further crosstalk among circadian rhythms, CIPN and other pain pathways. In addition to expression changes of canonical circadian genes, we identified broad circadian alteration of pain-related genes in both sites. On the other hand, paclitaxel treatment led to de novo diurnal expression of 547 and 1,055 transcripts in the DH and the DRG respectively (Fig. 5F). These results together highlight a strong reciprocal regulation between the circadian machinery and pain pathways. Of note, we found several families with strong circadian expression in the DRG and/or DH known to modulate pain response. The membrane transporter solute carrier (Slc) superfamily, ATP-dependent efflux ABC transporters and Ion channels (Kv, Nav, TRP) were identified as DEGs as a function of both circadian time and paclitaxel treatment, suggesting that these genes may form the physiological basis underlying the circadian oscillation of CIPN.

Chronotherapy, or the temporally-selective administration of treatments, has been shown to enhance the therapeutic index (efficacy vs. toxicity)^63,64^. Previously, in a clinical trial of oxaliplatin, fluorouracil, and folinic acid in colorectal cancer, chronotherapy had a significantly lower rate of peripheral neuropathy compared to constant-rate infusion (15% vs 31%, *p* < 0.01)^65^. Docetaxel therapeutic index was examined by administering docetaxel at 6 circadian time points and night time treatment showed better outcomes in antitumor efficacy and drug tolerance^66^. In a recent study, we demonstrated that sensitivity to multiple chemotherapy drugs is associated with expression of core clock genes^67^. Paclitaxel doses may be decreased by 20% to ameliorate a potential severe neuropathy, thus limiting its chemotherapeutic effects. Our current discovery of the circadian rhythm of paclitaxel-induced CIPN suggests a potential application of chronotherapy.

In conclusion, our study demonstrates a circadian regulation of CINP. The DRG harbors functional circadian oscillators with strong resilience against the cytotoxicity of paclitaxel. Global transcriptome profiling reveals a strong diurnal regulation of paclitaxel-induced transcriptomic landscape. Future mechanistic studies will investigate the crosstalk between circadian rhythms and the gene pathways identified, and its role in the temporal regulation of neuropathic pain.

## Methods

### Experimental animals

For CIPN studies, male adult Sprague–Dawley rats (200–350 g; Harlan Sprague Dawley Company, Houston, TX) were used as previously described^20^. They had free access to food and water and were housed in a room with a normal light–dark cycle (light cycle: 7:00 a.m. to 7:00 p.m.). All animals were habituated for 1 week before the experiments. The experimental protocol was approved by the Institutional Animal Care and Use Committee (IACUC) of the University of Texas MD Anderson Cancer Center.

For circadian bioluminescence and gene expression studies, the reporter mice or C57BL6/J wild-type mice were maintained and treated under IACUC guidelines, and the procedures were conducted as described in animal protocols approved by the IACUC of the University of Texas Health Science Center at Houston (UTHSC-H).

### Paclitaxel-induced neuropathic pain

Paclitaxel (GenDepot, Katy, TX) was dissolved in a vehicle solution (4% dimethyl sulfoxide and 4% Tween 80 in sterile saline) and was injected intraperitoneally in the morning (9 am-12 pm) at a dose of 2 mg/kg on days 0, 2, 4, and 6^68,69^. Control group rats were injected with the same volume of the vehicle without paclitaxel.

### Measurement of mechanical allodynia

To measure mechanical allodynia, we used the manual von Frey behavior test that has been described previously^70^. Briefly, rats were placed in a plastic chamber on top of a mesh screen, and the mechanical threshold of the left hind paw was determined by the up-down method^71^ using monofilaments (0.45–14.45 g). A filament was applied to the most sensitive parts of the paw’s plantar surface—the center of the paw or the base of the third or fourth toes—for 3–4 s. A sudden withdrawal of the foot during stimulation or immediately after removal of the filament was considered to be a positive response. The 50% threshold value was calculated from the pattern using the formula: 50% threshold = 10^(X+kd)^/10^4^, where X is the value of the final von Frey filament used in log units, k is the tabular value for the pattern of positive/negative responses, and d (0.22) is the mean difference between stimuli in log units^70^. After calculation, the threshold was expressed as Log value.

The investigator who conducted the behavioral tests was blinded to the vehicle or treatment status of the rats.

### Bioluminescence measurement from ex vivo DRG cultures

Mouse lumbar (L1–L5) DRG tissues were dissected with fine spring scissors and kept in chilled Hanks’ buffered salt solution (Invitrogen). Circadian bioluminescence measurement was performed as previously described^21^. Briefly, all dissected tissues were cultured on Millicell culture membranes (PICMORG50, Millipore) and were placed in 35 mm tissue culture dishes containing 2 mL DMEM media (Invitrogen) supplemented with 352.5 μg/ml sodium bicarbonate, 10 mM HEPES (Invitrogen), 2 mM l-Glutamine, 2% B-27 Serum-free supplement (Invitrogen), 25 units/ml penicillin, 25 μg/ml streptomycin (Invitrogen), and 0.1 mM luciferin potassium salt (L-8240, Biosynth AG). Sealed dishes were placed in a LumiCycle luminometer (Actimetrics, Wilmette, IL) and bioluminescence was recorded continuously. For the paclitaxel treatment group, 20 µM final concentration paclitaxel recording media or vehicle recording media were used to culture DRG. For data analysis, we used the LumiCycle data analysis program to calculate circadian period and amplitude (Actimetrics, Wilmette, IL).

### Immunohistochemistry of DRG

Freshly frozen DRG sections were briefly fixed with methanol for 10 min at − 20 °C then washed with PBS and Triton X-100 (PBS-t) three times. Sections were blocked in 10% normal goat serum for 60 min in PBS at room temperature and incubated with primary antibody diluted in 1% goat serum in PBS overnight at 4 °C. Slides were washed in PBS-t three times, then incubated with the secondary antibody for 60 min at room temperature, followed by three times of washes in PBS-t. Slides were stained with DAPI for 5 min, washed with PBS-t twice. Primary antibodies: BMAL1-GP^72^, 1:500 dilution; PER2-Rb^73^, 1:500 dilution; GFAP-Rb (Agilent Dako), 1:500 dilution; NeuN-Mouse (Millipore), 1:500 dilution. Secondary antibodies: anti-GP, anti-Rb AlexaFluor conjugates (ThermoFisher).

### Real-time qPCR and western blotting

RT-qPCR analysis was conducted as previously described^74,75^. Total RNA was extracted from frozen DRG by applying TRizol method (Invitrogen). One µg of extracted RNA were used for cDNA synthesis. Gene expression was analyzed by using QuantStudio 7 Flex Real-Time PCR. Immunoblotting was performed as described previously^74^ . Original, full-length immunoblots are presented in Fig. S7.

### Tissue preparation

Tissue was obtained at either *Zeitgeber* Time (ZT) ZT8 or ZT20 on day 27 after the first injection of paclitaxel or vehicle, with ZT0 corresponding to “light-on” during the 12:12 light:dark cycle. At either ZT8 (n = 3) or ZT20 (n = 3), the rats were anaesthetized deeply with 4% isoflurane for induction for 5 min and then 3% for maintenance. Under anesthesia, rat hair was removed over the thoracic region, and thoracotomy was performed, and the rat was perfused with cold saline. The L1–6 DRG and DH were removed, frozen in liquid nitrogen, and stored at − 80 °C. Total RNA was extracted from frozen DRG and DH tissues with TRIzol (Invitrogen).

### RNA sequencing analysis

Two micrograms of extracted RNA samples from three rats were used for library construction and RNA sequencing analysis (Novogene). PolyA enriched non-stranded RNA sequencing was carried out by Novogene (US) on Illumina HiSeq 2500 with 150-bp paired-end reads. After adaptor sequences were trimmed using Trimmomatic^76^, the sequence reads were mapped to the rat genome (RGSC 6.0/rn6) using STAR^77^. To obtain reliable alignments, the reads with a mapping quality of less than 10 were removed by SAM tools^78^. Ensemble annotation (41,078 transcripts) was used for gene annotation, and the fragments mapped to the exons were quantified using Homer^79^. We assumed that a gene was expressed if there were more than 20 fragments mapped in the exons of the gene on average of samples in DH or DRG. For differentially expressed gene analysis, the longest transcript of each gene was selected and the low-expression genes were filtered out with a cut-off of 0.5 Fragments Per Kilobase of exon per Million mapped fragments (FPKM). The differentially expressed genes, with thresholds of *p* < 0.05 and fold change > 1.2, were determined by using DESeq2^80^. Functional enrichment analysis was carried out using Metascape^25^.

### Statistical analyses

Data were summarized as means with standard errors of the means for the behavioral testing. The data were analyzed in the GraphPad Prism 6 by one-way or two-way ANOVA, followed by the Tukey Post-hoc test for behavioral testing and the Mann–Whitney U test for RNA sequencing. In all cases, *p* < 0.05 was considered statistically significant.

## Supplementary information

Supplementary file 1

Supplementary Table S1.

Supplementary Table S2.

Supplementary Table S3.

Supplementary Table S4.

Supplementary Table S5.

Supplementary Table S6.

Supplementary Table S7.

Supplementary Table S8.

Supplementary Table S9.

Supplementary Table S10.

## Data Availability

All raw data associated with this article are available upon reasonable request.
