# Supplementary material for: Circadian regulation of chemotherapy-induced peripheral neuropathic pain and the underlying transcriptomic landscape
Source: Sci Rep. 2020 Aug 14;10:13844. doi: 10.1038/s41598-020-70757-w (PMC7427990; doi:10.1038/s41598-020-70757-w)
Supplement: Supplementary file 1 [file 41598_2020_70757_MOESM1_ESM.pdf]

**Supplementary Information**

# Circadian regulation of chemotherapy-induced peripheral neuropathic pain and the underlying transcriptomic landscape

Hee Kee Kim[1,8], Sun-Yeul Lee[2,3,8], Nobuya Koike[4], Eunju Kim[2], Marvin Wirianto[2], Mark J. Burish[5], Kazuhiro Yagita[4], Hyun Kyoung Lee[6], Zheng Chen[2], Jin Mo Chung[7], Salahadin Abdi[1], Seung-Hee Yoo[2§]

1. Department of Pain Medicine, Division of Anesthesiology, Critical Care and Pain Medicine, The University of Texas MD Anderson Cancer Center, Houston, TX 77030, USA
2. Department of Biochemistry and Molecular Biology, The University of Texas Health Science Center at Houston, 6431 Fannin St., Houston, TX 77030, USA
3. Department of Anesthesiology and Pain Medicine, Chungnam National University Hospital, Daejeon, South Korea
4. Department of Physiology and Systems Bioscience, Graduate School of Medical Science, Kyoto Prefectural University of Medicine, Kyoto, Japan
5. Department of Neurosurgery, The University of Texas Health Science Center at Houston, 6400 Fannin St., Houston, TX 77030, USA
6. Department of Pediatrics, Baylor College of Medicine, Neurological Research Institute, Texas Children's Hospital, Houston, Texas 77030, USA
7. Department of Neuroscience, Cell Biology and Anatomy, University of Texas Medical Branch, Galveston, TX, USA
8. These authors contributed equally to this work.

[§]: To whom correspondence should be addressed. E-mail: seung-hee.yoo@uth.tmc.edu

Supplementary Information for the above manuscript contains seven supplementary figures (Fig. S1-S7) and ten supplementary tables (Table S1-S10).

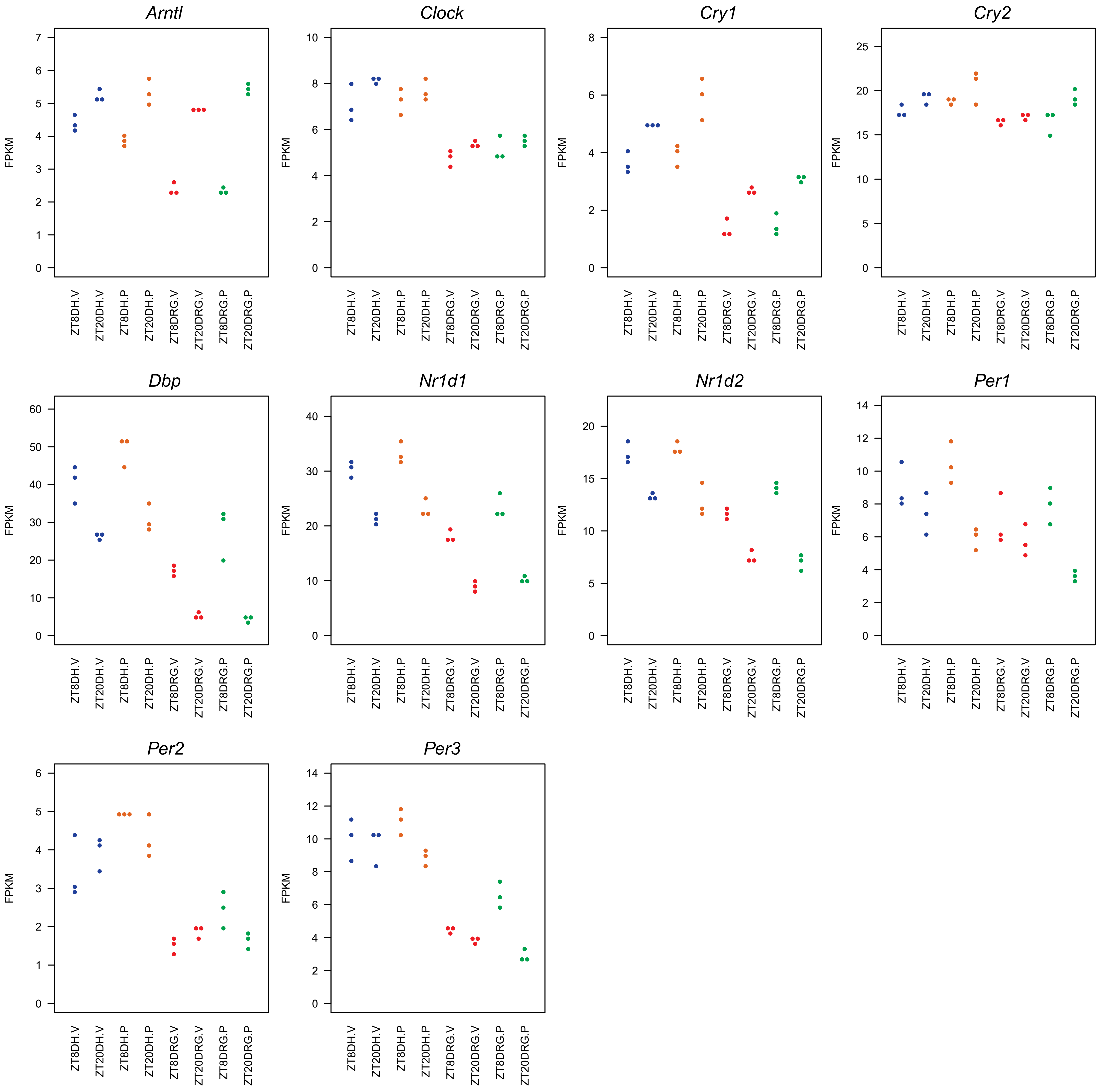


**Fig. S1.** Expression of core clock genes in the DH and the DRG.

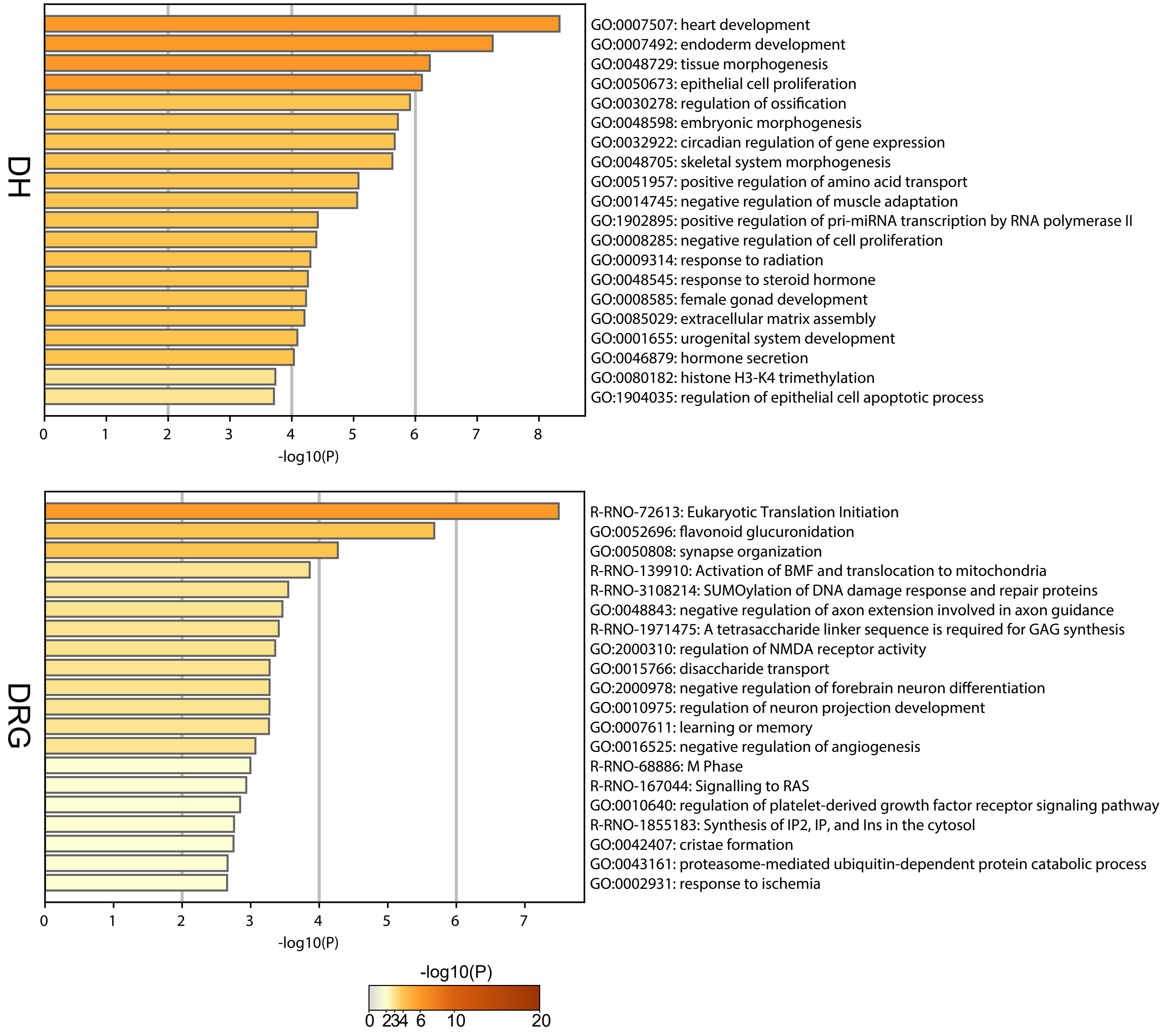


**Fig. S2.** Heat map showing the top enrichment clusters by Metascape analysis using paclitaxel-induced de novo diurnal DEGs in the DH and the DRG.

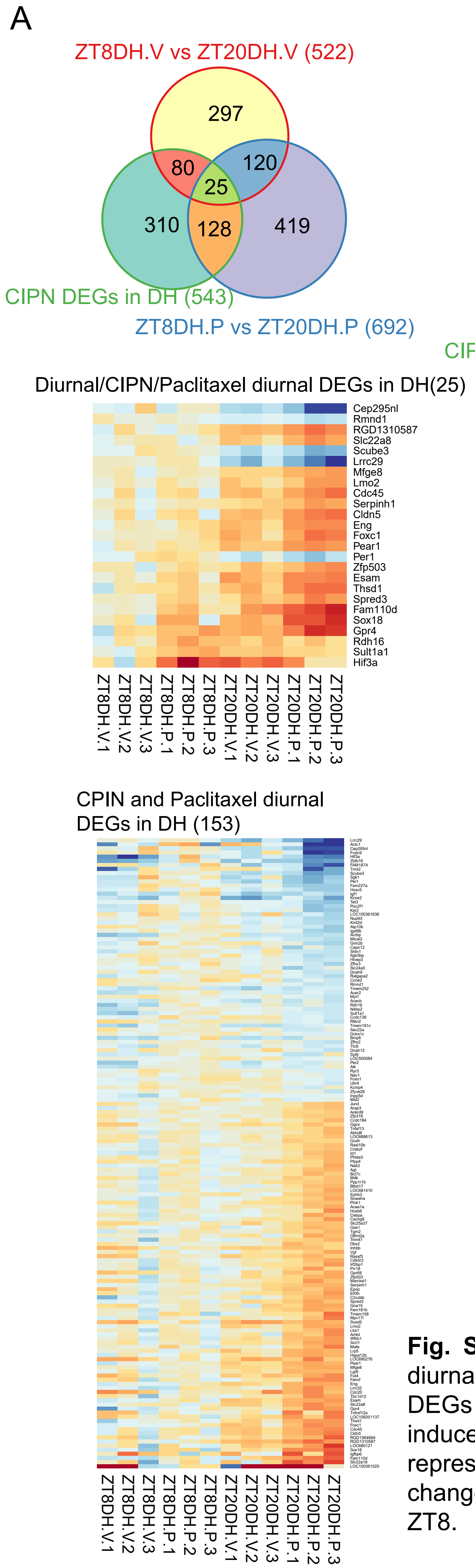

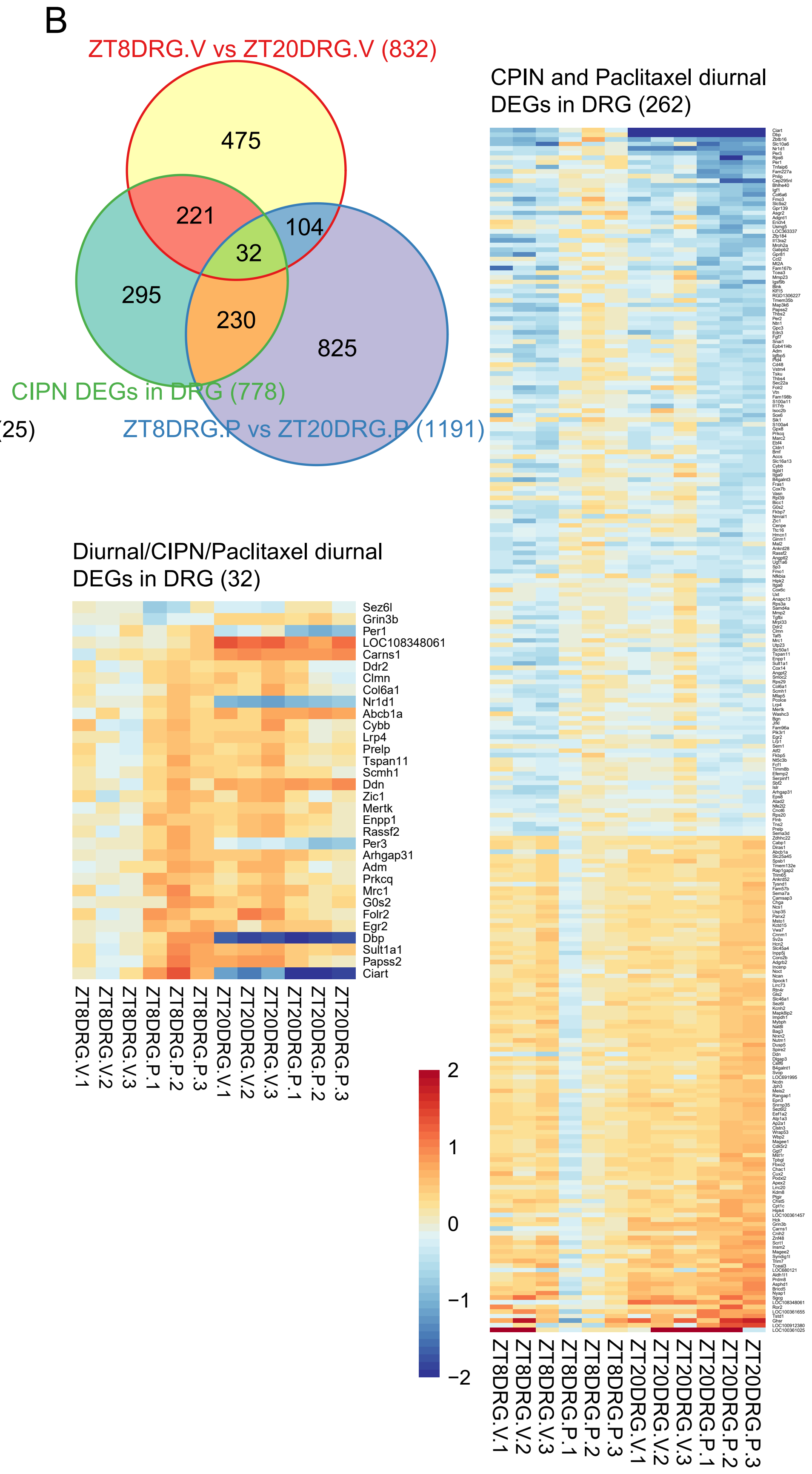


**Fig. S3.** Venn diagrams of diurnal, CIPN, and paclitaxel-induced diurnal DEGs in the DH (A) and the DRG (B). Heat maps indicate DEGs at the intersections of diurnal genes, CIPN, and paclitaxel-induced diurnal DEGs in the DH and the DRG. Each gene is represented as a horizontal line ordered vertically by log2 fold change in expression level at ZT20 with vehicle treatment relative to ZT8.

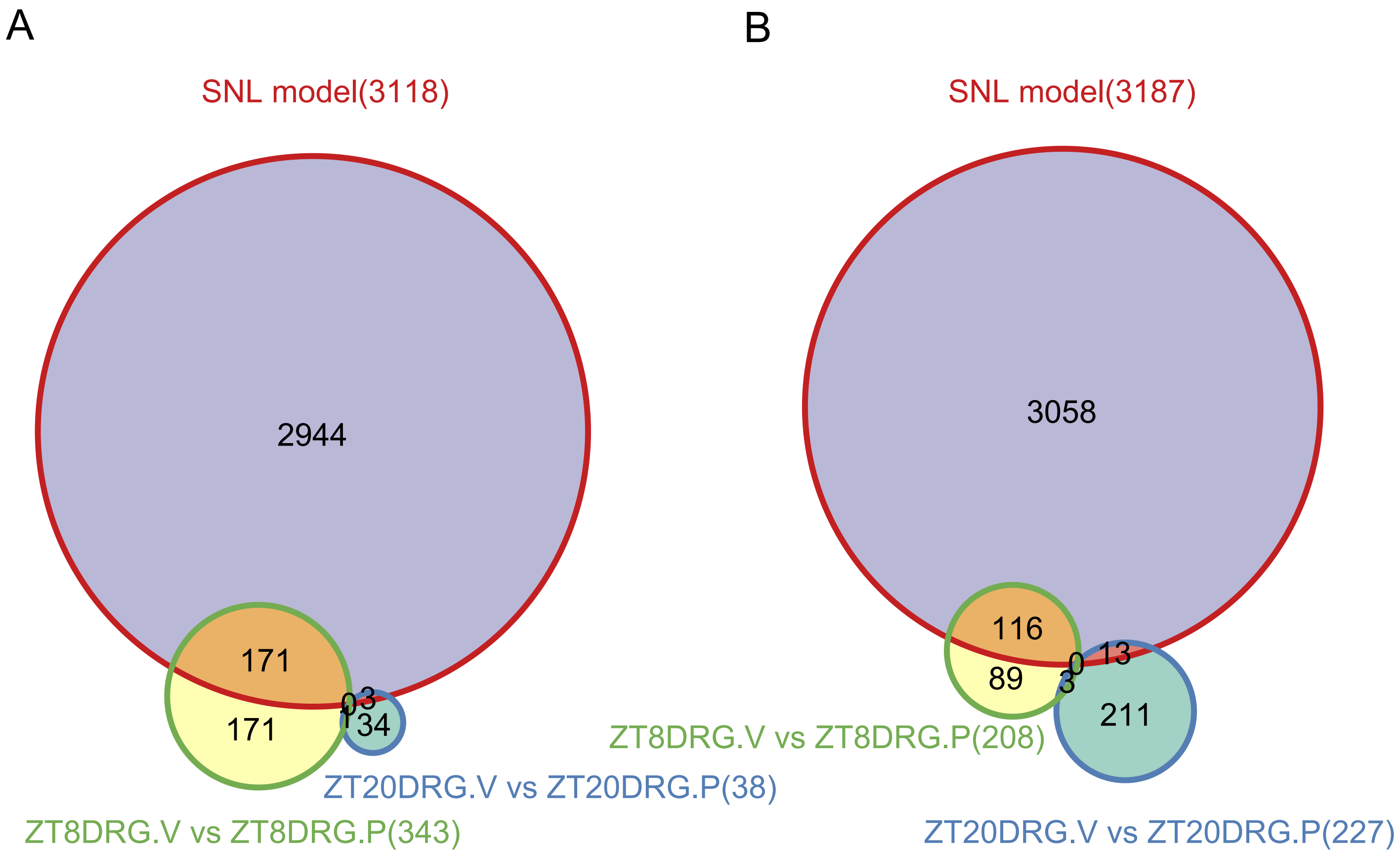


**Fig. S4.** Venn diagrams of DEGs of SNL and CIPN. (A) Up-regulated genes from both SNL and paclitaxel CIPN models. (B) Down-regulated genes from both SNL and paclitaxel CIPN models.

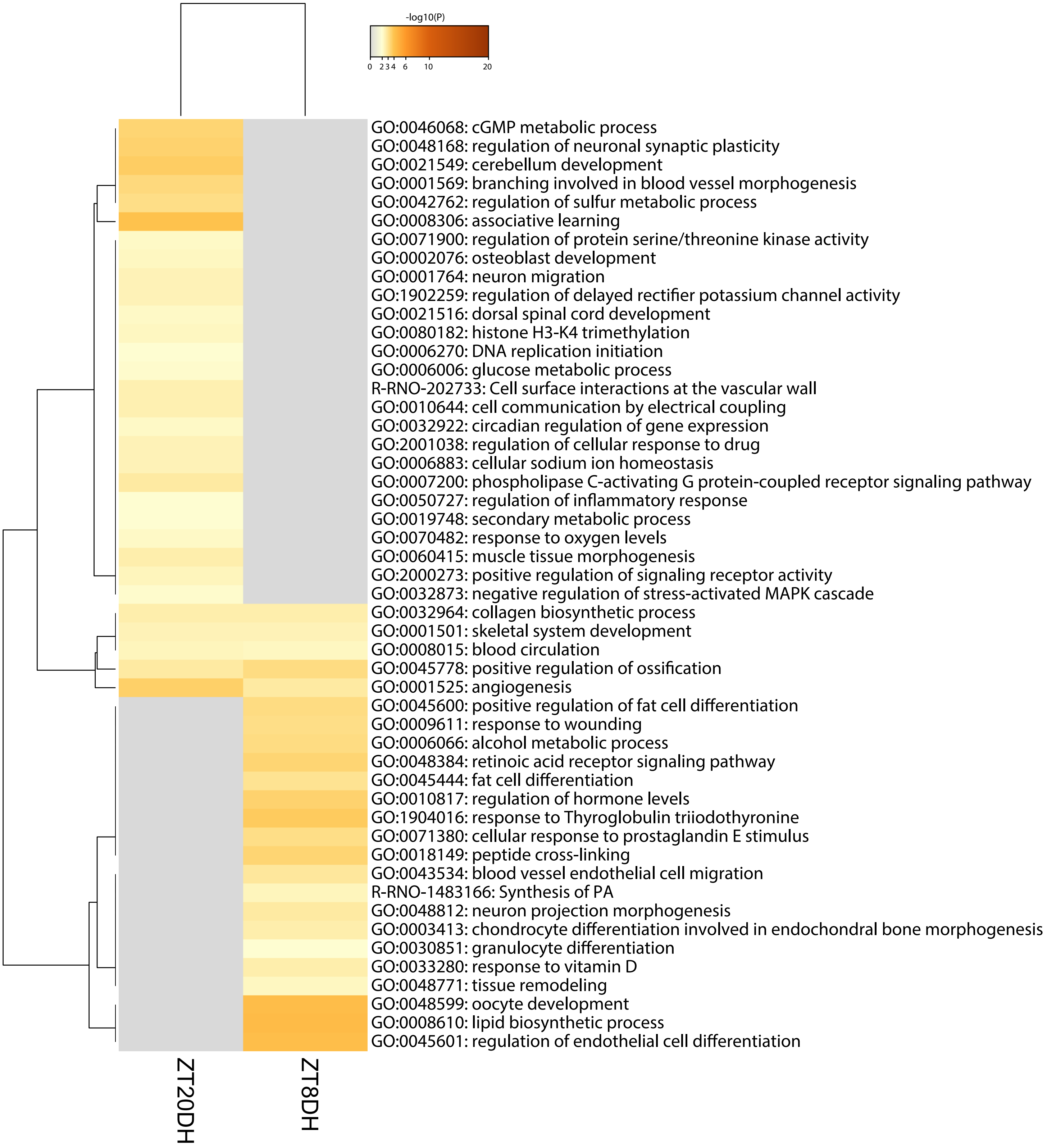


**Fig. S5.** Heat map showing the top 50 enrichment clusters by Metascape analysis using paclitaxel-induced DEGs in the DH at two circadian time points (ZT8 and ZT20).

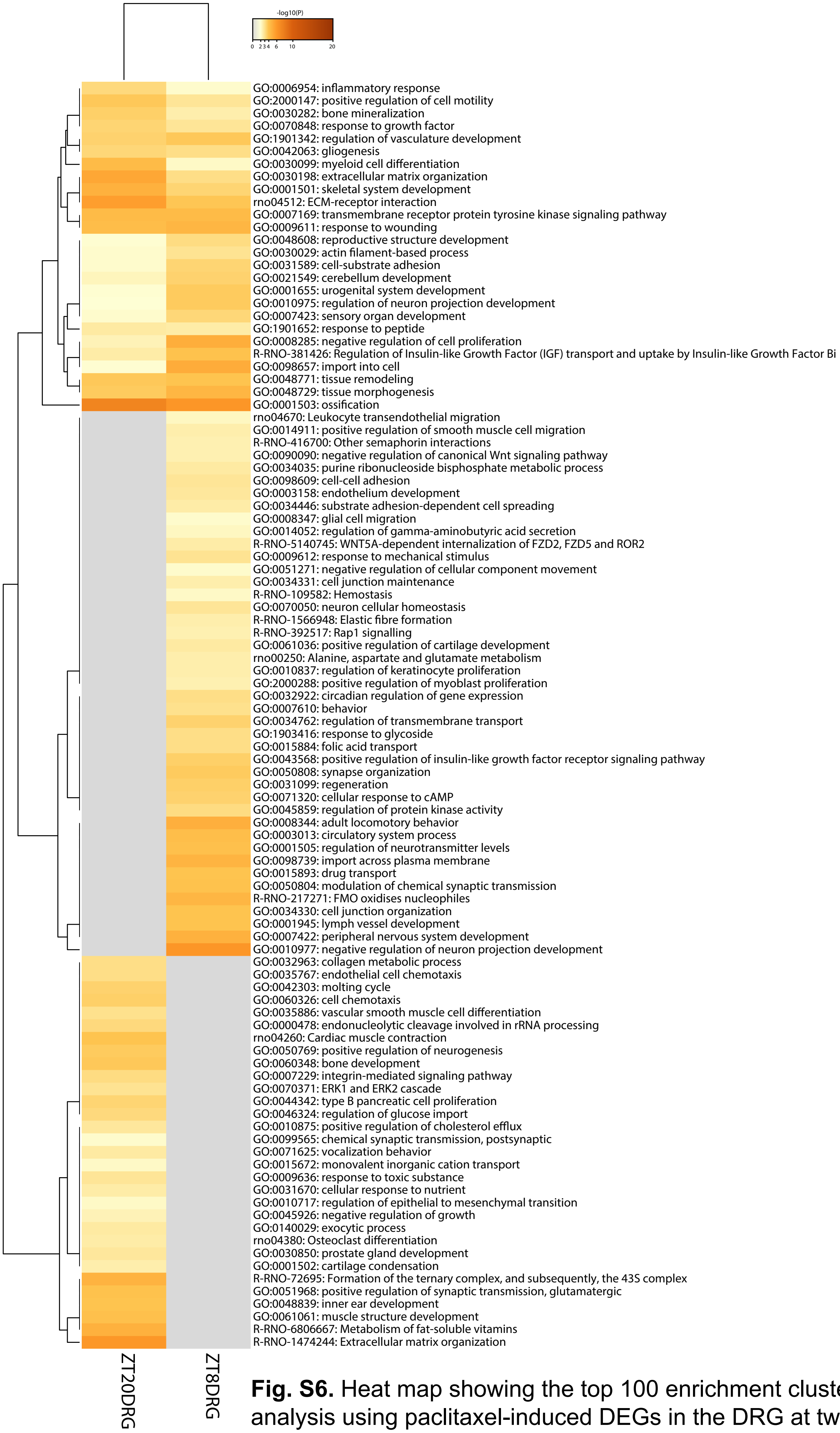


**Fig. S6.** Heat map showing the top 100 enrichment clusters by Metascape analysis using paclitaxel-induced DEGs in the DRG at two circadian time points (ZT8 and ZT20).

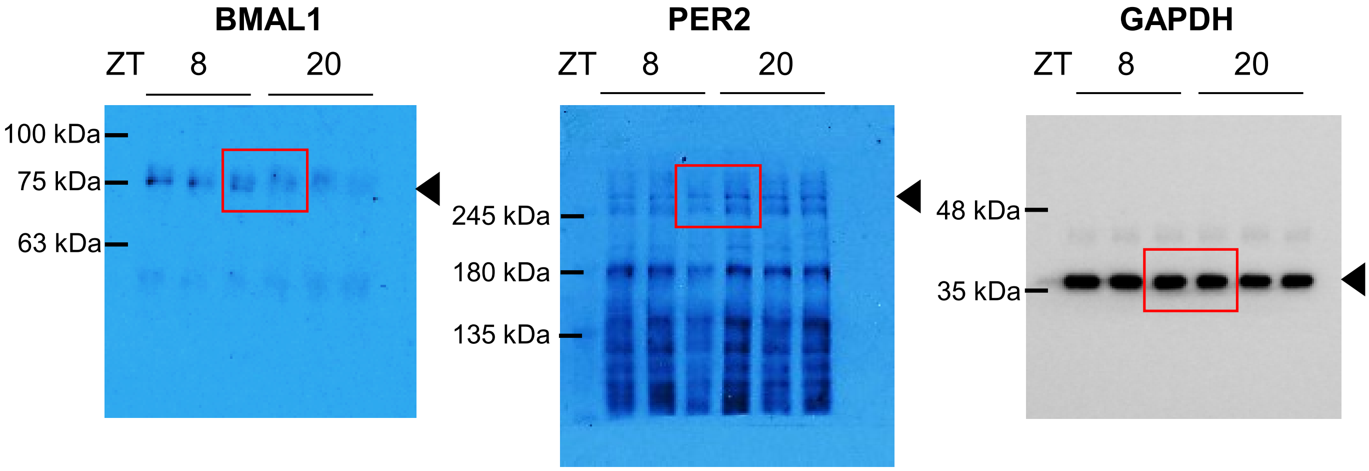


**Fig. S7.** Original, full-length immunoblots for Fig. 4D.
